# *A^1v^B* Genotype With Weak A Antigen Expression: Resolution of an ABO Discrepancy Using Serological and Molecular Approaches

**DOI:** 10.1002/kjm2.70192

**Published:** 2026-03-02

**Authors:** Wan‐Hua Yang, Fen‐Cing Liou

**Affiliations:** ^1^ Department of Pathology and Laboratory Taipei Veterans General Hospital Hsinchu Branch Hsinchu Taiwan; ^2^ Department of Industrial Engineering and Management Yuan Ze University Taoyuan Taiwan; ^3^ Department of Medical Laboratory Science and Biotechnology Yuanpei University of Medical Technology Hsinchu Taiwan

ABO blood group discrepancies caused by weak A subgroups remain a recognized challenge in routine transfusion practice, particularly in elderly patients requiring urgent blood support [1]. We report an ABO discrepancy identified during pre‐transfusion testing in a 96‐year‐old male admitted for gastrointestinal hemorrhage and severe anemia, with a hemoglobin level of 6.5 g/dL at presentation. Routine ABO forward grouping demonstrated mixed‐field agglutination with anti‐A and a strong reaction with anti‐B, while reverse grouping showed no agglutination with either A_1_ or B reagent cells. RhD typing was positive, and antibody screening as well as auto‐control testing yielded negative results.

Because the initial serological findings were inconclusive, further investigations were undertaken to clarify the patient's ABO status. Adsorption–elution testing was performed using polyclonal anti‐A reagent incubated with the patient's red blood cells at 4°C, followed by heat elution. The resulting eluate reacted strongly (3+) with A_1_ reagent cells but showed no reactivity with B or O cells, confirming the presence of weak A antigen expression on the red blood cells. There was no history of recent transfusion, hematopoietic stem cell transplantation, or clinical evidence suggestive of chimerism, making these alternative causes of mixed‐field agglutination unlikely in this case.

To investigate the molecular basis of the discrepancy, polymerase chain reaction–restriction fragment length polymorphism (PCR‐RFLP) analysis targeting *ABO* exons 6 and 7 was performed. Digestion of exon 6 with *Kpn I* revealed an intact 350 bp fragment, indicating the absence of the c.261delG mutation and effectively excluding the common *O* allele [1]. Digestion of exon 7 with *Hpa II* produced a restriction fragment pattern (309, 223, 204, 150, 119 bp) characteristic of the *A* ^*1v*^ allele [2]. In conjunction with the serological demonstration of B antigen expression, the patient's genotype was interpreted as *A* ^*1v*^ *B* (Figure 1).

**FIGURE 1:**
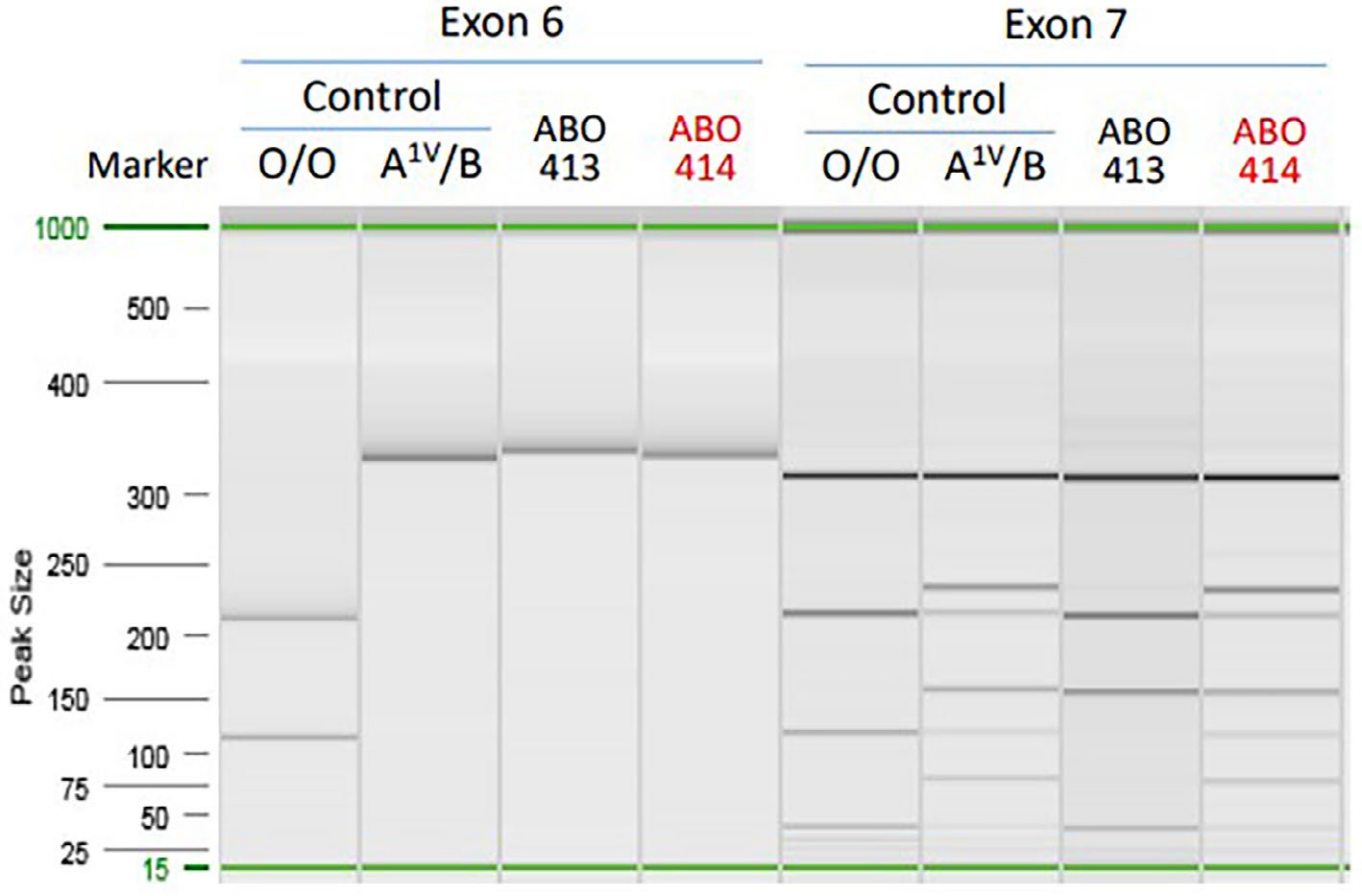
PCR‐RFLP analysis of *ABO* gene. Lane 1: DNA Ladder (marker). Lane 2: Patient Sample—Exon 6 digested with *Kpn* I (350 bp band, indicating no *O* deletion). Lane 3: Patient Sample—Exon 7 digested with *Hpa* II (Fragments: 309, 223, 204, 150, 119 bp).

The *A* ^*1v*^ allele (ABO*A^1^.02) is the predominant *A* allele in East Asian populations [3] and is generally associated with normal A_1_ antigen expression. Previous molecular studies have shown that weak A phenotypes identified in individuals with an A^1v^ backbone are more commonly the result of additional point mutations, regulatory changes, or hybrid alleles rather than the A^1v^ backbone itself [4]. The findings in the present case are consistent with this observation, as weak A antigen expression was detected despite an *A* ^*1v*^‐associated restriction pattern. Although PCR‐RFLP cannot detect rare sequence variants or complex hybrid structures, it remains a practical and accessible molecular tool for identifying major *ABO* allele backbones in routine laboratory settings.

From a transfusion management perspective, the patient received group O red blood cells and group AB plasma in accordance with established recommendations for unresolved or weak ABO phenotypes. No transfusion‐related adverse events were observed. This case underscores the value of combining detailed serological investigation with targeted molecular analysis to support safe transfusion decisions when comprehensive sequencing methods are not readily available.

## Funding

This study was supported by a grant (2026‐VHCT‐RD‐P002) from Taipei Veterans General Hospital Hsinchu Branch.

## Conflicts of Interest

The authors declare no conflicts of interest.

## Data Availability

The data that support the findings of this study are available from the corresponding author upon reasonable request.
